# Interaction of *Salmonella* Gallinarum and *Salmonella* Enteritidis with peripheral leucocytes of hens with different laying performance

**DOI:** 10.1186/s13567-021-00994-y

**Published:** 2021-09-25

**Authors:** Sravya Sreekantapuram, Christian Berens, Stefanie A. Barth, Ulrich Methner, Angela Berndt

**Affiliations:** 1 Research Group Microbial Immunology, Leibniz Institute for Natural Product Research and Infection Biology – Hans Knoell Institute, Jena, Germany; 2 Institute of Molecular Pathogenesis, Friedrich-Loeffler-Institut, Jena, Germany; 3 Institute of Bacterial Infections and Zoonoses, Friedrich-Loeffler-Institut, Jena, Germany

**Keywords:** chicken lines, immune response, *Salmonella* Enteritidis, *Salmonella* Gallinarum, whole blood

## Abstract

*Salmonella enterica* ssp. *enterica* serovars Enteritidis (SE) and Gallinarum (SG) cause different diseases in chickens. However, both are able to reach the blood stream where heterophils and monocytes are potentially able to phagocytose and kill the pathogens. Using an ex vivo chicken whole blood infection model, we compared the complex interactions of the differentially host-adapted SE and SG with immune cells in blood samples of two White Leghorn chicken lines showing different laying performance (WLA: high producer; R11: low producer). In order to examine the dynamic interaction between peripheral blood leucocytes and the *Salmonella* serovars, we performed flow cytometric analyses and survival assays measuring (i) leucocyte numbers, (ii) pathogen association with immune cells, (iii) *Salmonella* viability and (iv) immune gene transcription in infected whole blood over a four-hour co-culture period. Inoculation of blood from the two chicken lines with *Salmonella* led primarily to an interaction of the bacteria with monocytes, followed by heterophils and thrombocytes. We found higher proportions of monocytes associated with SE than with SG. In blood samples of high producing chickens, a decrease in the numbers of both heterophils and *Salmonella* was observed. The *Salmonella* challenge induced transcription of interleukin-8 (IL-8) which was more pronounced in SG- than SE-inoculated blood of R11. In conclusion, the stronger interaction of monocytes with SE than SG and the better survivability of *Salmonella* in blood of low-producer chickens shows that the host–pathogen interaction and the strength of the immune defence depend on both the *Salmonella* serovar and the chicken line.

## Introduction

*Salmonella enterica* subsp. *enterica* (*S.*) belong to the family of *Enterobacteriaceae* and are facultative intracellular bacteria with the potential to cause infections in both humans and animals. More than 2600 different serovars have been described so far, which have been subdivided into three types of host adaptation, i.e. (i) ubiquitous serovars occurring in a wide range of hosts (non-host-specific serovars), for example *Salmonella* Enteritidis (SE) or Typhimurium, (ii) host-adapted *Salmonella* serovars and (iii) host-restricted serovars, such as *Salmonella* Gallinarum (SG), which are almost exclusively associated with a particular host species [1].

The host-restricted SG causes fowl typhoid in chickens, which is characterised by a primarily systemic infection with little or no initial intestinal involvement. This typhoidal infection is generally associated with high mortality in chickens of all ages. Surviving chickens can remain carriers for the rest of their lives [2]. In Europe, the disease has been almost entirely eradicated but remains a particular endemic issue in Asia and South America [3]. The avian immune response against SG is still poorly understood in chickens. Three phases of avian systemic salmonellosis, however, have already been described [4], each characterised by a significant interaction with the immune system. In the first phase, the bacteria enter the body presumably via enterocytes, Peyer’s patches and caecal tonsils [5]. Within the intestinal mucosa, an inflammatory response with a significant influx of heterophils is absent [6]. In the second phase of illness, mucosal macrophages and dendritic cells engulf the bacteria and transport them into other organs of the body [2]. This is accompanied by severe systemic disease and bacteremia. The third phase features the elicited immune response with the production of *Salmonella* specific antibodies and T-cell proliferation [7].

The non-host-adapted SE typically causes an extensive intestinal infection with varying degrees of systemic dissemination in poultry. Chicks younger than 3 days are very susceptible and severe illness with blood stream infection is possible. Older chickens are more resistant [8]. A *Salmonella* infection in poultry entails a potential risk for human health. Non-typhoidal *Salmonella*-contaminated poultry products, such as chicken meat, eggs, and egg products, remain the main source for human salmonellosis. SE still represents the most important cause of human *Salmonella* infection in the European Union [9].

In contrast to SG, the immune response against non-host-specific serovars, especially SE, has been intensively investigated in chickens. The immune reaction in caecum, spleen, bursa of Fabricius and blood is characterised by a significant influx of all sorts of immune cells [10, 11]. Heterophils, the avian counterparts of mammalian neutrophils, are considered crucial in the initial effector response of young poultry [12–14] and macrophages are important in regulating the progress of innate and the development of adaptive immune responses [15].

To prevent *Salmonella* infection in chickens, which is a prerequisite to reduce exposure for human beings, it is not only of interest to apply more effective hygiene regimes in poultry production units, but also to create new vaccine strategies and/or to utilize chicken lines with higher intrinsic resistance. To develop alternative strategies against *Salmonella* in poultry, however, a detailed understanding of host–pathogen interactions and the host’s immune response to the infection is an essential prerequisite.

Comparative work on immune reactions against typhoidal and non-typhoidal serovars is generally rare and has mostly been done with cell lines [16] or isolated cells [17, 18]. Although both *Salmonella* serovars, SE and SG, are able to enter the blood stream, nothing is known about their survival in this unique environment as well as on their interplay with the immune cells therein. For that reason, we compared SE and SG with respect to their dynamic host–pathogen interaction within the complex milieu of avian peripheral blood of two differently performing laying hen lines.

## Materials and methods

### Animals

White Leghorn chickens of two lines possessing different egg laying performances and genetic backgrounds were used: high-performing white layers (WLA) and low-performing white layers (R11). The chicken lines have been described earlier [19]. WLA originates from a breeding line of Lohmann Tierzucht GmbH, Cuxhaven. The White Leghorn line R11 descends from the Cornell Line K [20] and has been managed as conservation flock at the Friedrich-Loeffler-Institut (FLI) since 1965. Chicks were hatched from the eggs (kindly provided by Prof. Steffen Weigend; FLI, Institute of Farm Animal Genetics) and housed at FLI facilities (Institute of Molecular Pathogenesis, Jena, Germany). The animal housing in floor management was in accordance with the guidelines for animal welfare set forth by the European Community. Throughout the study, the chickens were reared and kept under standard conditions at a room temperature of 18–20 °C and a relative humidity of 50–60%. Commercial feed in powder and pellet form (without antibiotics or other additives) and drinking water were both available ad libitum. The study was done in strict accordance with the German Animal Welfare Act. The protocol was approved by the Committee on the Ethics of Animal Experiments and the Protection of Animals of the State of Thuringia, Germany (registration number: 04-001-14). Six animals, aged 16 to 19 months, from each chicken line (in total 12 animals) were used for the experiments conducted in this study.

### Bacteria

Strains of *Salmonella* Enteritidis (SE147; SE) [21] and *Salmonella* Gallinarum (SG9; SG) [22] were used for ex vivo inoculation of avian blood (Table 1). To generate the GFP-expressing *Salmonella* strains, the plasmid pUC19Pcatgfp+, constitutively expressing the GFP-variant GFP+ [23], was used [24]. The restriction-deficient, modification-proficient *Salmonella* Typhimurium strain LB5000 (LT2-derived; r_LT_^–^, m_LT_^+^, r_SA_^–^, m_SA_^+^, r_SB_^–^, m_SB_^+^, *metA22*, *metE551*, *trpD2*, *leu*; [25] was first transformed chemically with the plasmid. Chemically competent cells of the *Salmonella* strains SE and SG were then transformed with pUC19Pcatgfp+ isolated from *S*. Typhimurium LB5000.

**Table 1:** ***Salmonella* strains used for this study**

*Salmonella* serovar (strain)	Group	Antigenic formula	Phage type	Virulence-associated plasmid	Reference
Enteritidis (SE147)	D_1_	1,9,12:[f],g,m,[p] [1, 7]:	PT4	37 MDa	[51]
Gallinarum (SG9)	D_1_	1,9,12:-:-	none	85 kb	[28]

GFP-expressing SE and SG were cultivated overnight at 37 °C and 180 rpm (VXR basic Vibrax (IKA, Staufen, Germany) in nutrient broth (SIFIN, Berlin, Germany). The overnight culture was inoculated 1:100 into fresh nutrient broth and incubated at 37 °C and 180 rpm until OD_600_ 0.6–0.7 was reached. The cultures were then washed thrice in phosphate-buffered saline (PBS). The doses for the bacterial strains were obtained based on their OD_600_-cfu correlation. Cultures were diluted to the desired concentrations with PBS before inoculation of whole blood.

### Whole blood ex vivo infection assay

The whole-blood infection assay was performed as described [24]. Six chickens per line (in total 12 animals) were used. Blood samples from two to four animals (one or two from each line) were tested on the same day. Briefly, peripheral blood was drawn by wing venipuncture (*Vena ulnaris*) into commercial hirudin-coated syringes (S-Monovette®, 2.7 mL Hirudin, Sarstedt, Germany). Hirudin was chosen as anticoagulant as it was previously shown to have no effect on thrombocytes and complement activation [24, 26]. The number of 10^6^ GFP-expressing microbial cells was added to 1 mL of blood. Samples were incubated at 41 °C, 5% CO_2_ under constant rotation (20 rpm, Tube rotator, VWR, Darmstadt, Germany, product number VWRI444-0500) for 240 min. Every 30 min, a volume of blood was taken for flow-cytometric analysis (20 µL), determining the colony forming units of the pathogens (cfu, 10 µL) and for establishing the transcription levels of selected immune-related genes by RT-PCR (50 µL; 0, 30, 90, 150 and 240 min after inoculation of bacteria).

### Bacterial growth in blood

To determine survival and growth of the pathogens in avian whole blood, serial dilutions were plated on blood agar plates in 2–4 technical replicates. The cfu per time point was determined by counting the colonies and calculated in accordance to the dilution after 16–20 h incubation at 37 °C.

### Flow cytometry

To examine both the number of immune cells in blood samples and their interaction with the pathogens, flow cytometric analysis was performed on the basis of recently described protocols [24, 27]. Briefly, 20 µL of the cultured whole blood was added to 980 µL phosphate-buffered saline (PBS). 50 µL of the diluted blood was added to 20 μL of an antibody mixture placed previously into a True-Count Tube (Becton Dickinson GmbH). The antibody mixture contained the following monoclonal antibodies: the monocyte/macrophage marker KUL01-RPE (clone KUL01), the macrophage/thrombocyte marker K1-PE (clone K1), the leucocyte marker CD45-APC (clone LT40) and the T cell marker CD3-PE-Cy5 (clone CT-3) (with exception of K1, all from Southern Biotechnology Associates; Eching, Germany). The K1 antibody (kind gift of Prof. Bernd Kaspers, Ludwig-Maximilians-University Munich) was conjugated with the R-Phycoerythrin conjugation kit (Abcam, Cambridge, UK) according to the manufacturers’ instructions. Prior to the study, optimal antibody concentrations were tested and appropriate fluorescence-minus one controls performed. Additional isotype controls (AbDSerotec) were used to confirm antibody binding specificity.

**Figure 1:**
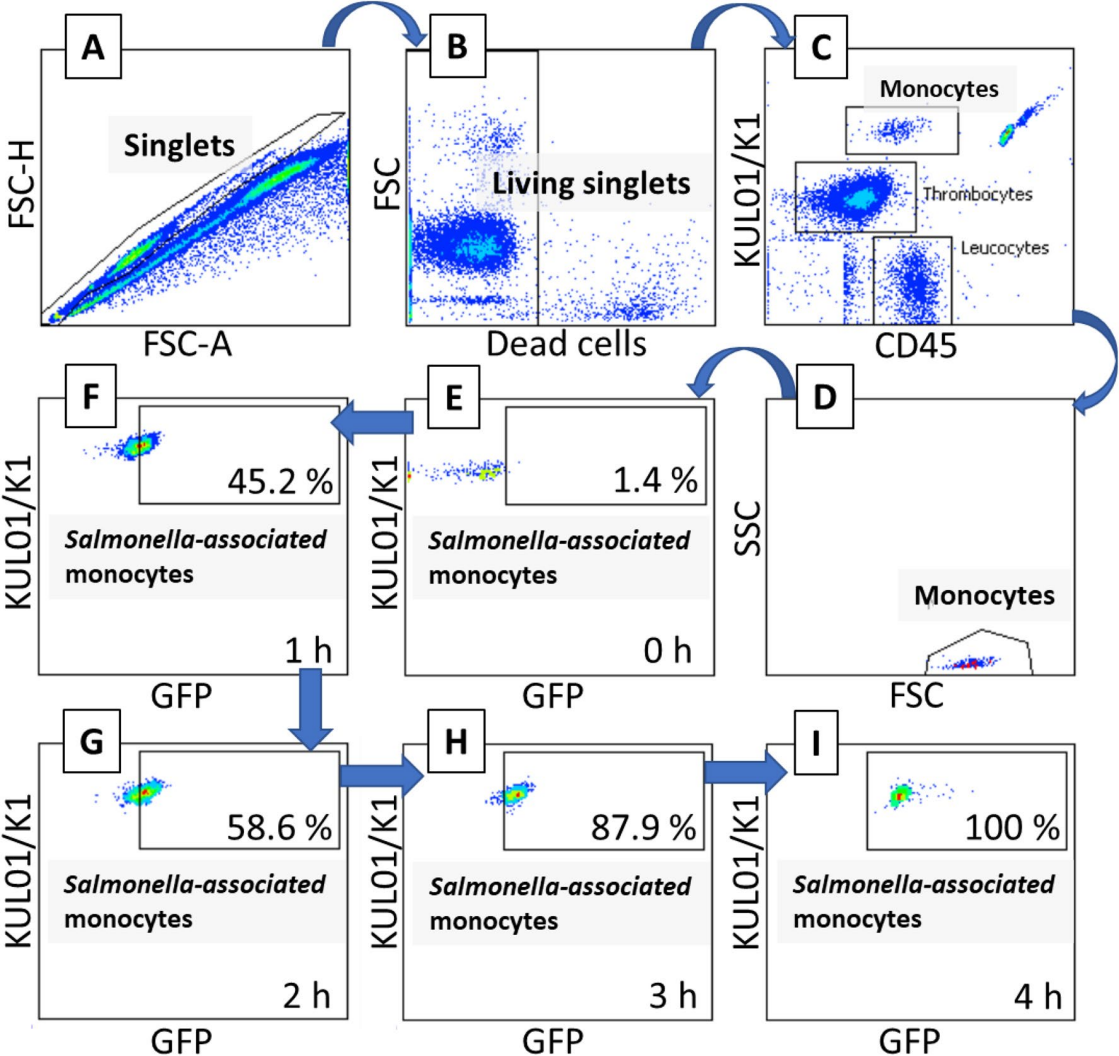
**Representative gating strategy used for monocyte identification and example for monocyte association with SE.** After excluding of doublets and dead cells, monocytes were gated from the dot-plot diagram showing CD45-positive leucocytes and KUL01-positive monocytes and subsequently back-gated using the FSC-SSC dot plot (**A–D**). Then, monocytes were analysed in regard to their association with GFP-transformed *Salmonella* by means of a dot plot showing the KUL01-positive monocytes and the green-fluorescent SE at different time points after *Salmonella* inoculation of the blood samples (**E–I**).

Samples were incubated for 45 min in the dark. Thereafter, 300 μL of PBS as well as DAPI (1 µg/mL PBS; Sigma, Taufkirchen, Germany) were added to the sample. After 2 min incubation, leucocyte counts were acquired by use of a BD FACSCanto II flow cytometer (Becton Dickinson, Heidelberg, Germany) equipped with a 488 nm, 633 nm and 405 nm laser. Up to 40 000 beads were recorded and stored together with the immune cells and *Salmonella* of each sample, for absolute quantification of the cell populations. The analysis was performed by use of the BD FACSDiva software (Version 6.1.3, BD Biosciences) as described [24]. At first, living single cells were selected by their negative DAPI staining. Then, thrombocytes and monocytes were selected from the dot plot K1/KULO1 against CD45 (Figure 1). The CD45-positive leucocytes were used to separate CD3-positive T lymphocytes. Heterophils were selected by back gating from the dot plot of CD45^+^ against SSC [27].

Each selected leucocyte subset was analysed with respect to its percentage of cells associated with the GFP-expressing pathogens. In addition, the GFP-expressing pathogens were identified by using the FITC channel and sub-gated against the immune cell-specific markers to obtain the percentage of pathogens interacting with the different immune cells (Figure 1).

For calculation of absolute immune cell numbers, all single populations were back-gated against FSC/SSC. Absolute numbers of blood cells were calculated using the following equation:

$$\frac{{\text{absolute cell count}}}{{\mu {\text{L of blood}}}} = \frac{{\text{cells counted}}}{{\text{beads counted}}} \times \frac{{\text{total content of beads per tube}}}{{\text{blood volume per tube}}}$$

### RNA extraction and quantitative real-time reverse transcription (RT)-PCR

To analyse the transcription of important immune-related genes, total RNA was extracted from 50 µL blood/sample using the RNeasy Mini Kit for blood (Qiagen, Hilden, Germany) according to the manufacturer’s protocol. The QuantiTect SYBR Green real-time one-step RT-PCR kit (Qiagen) and avian-specific primers for IL-1b, IL-6, the chemokine IL-8 (CAF,CXCLi2), the effector iNOS and the transcription factor LITAF (lipopolysaccharide-induced TNF-alpha factor) were used as described [24] to determine mRNA expression rates. Efficiencies of the primers had been tested prior to the study. The expression was normalised to the house keeping gene glycerinaldehyde-3-phosphate (GAPDH). Results are given as 40-ΔCt values.

### Statistical analyses

Six independent biological replicates derived from different animals were used for the experiments. Data are represented as arithmetic mean ± SD. Data were normally distributed as demonstrated by the Kolmogorov Smirnov test in GraphPad Prism 7. Data were analysed by 2-way ANOVA followed by Tukey’s multiple comparison test (GraphPad Prism 7) to compare inoculated and non-inoculated samples, different time points, different pathogens and different chicken lines. *P* values ≤ 0.05 were considered significant.

## Results

### Effects of *Salmonella* infection on the viability of leucocytes

**Figure 2:**
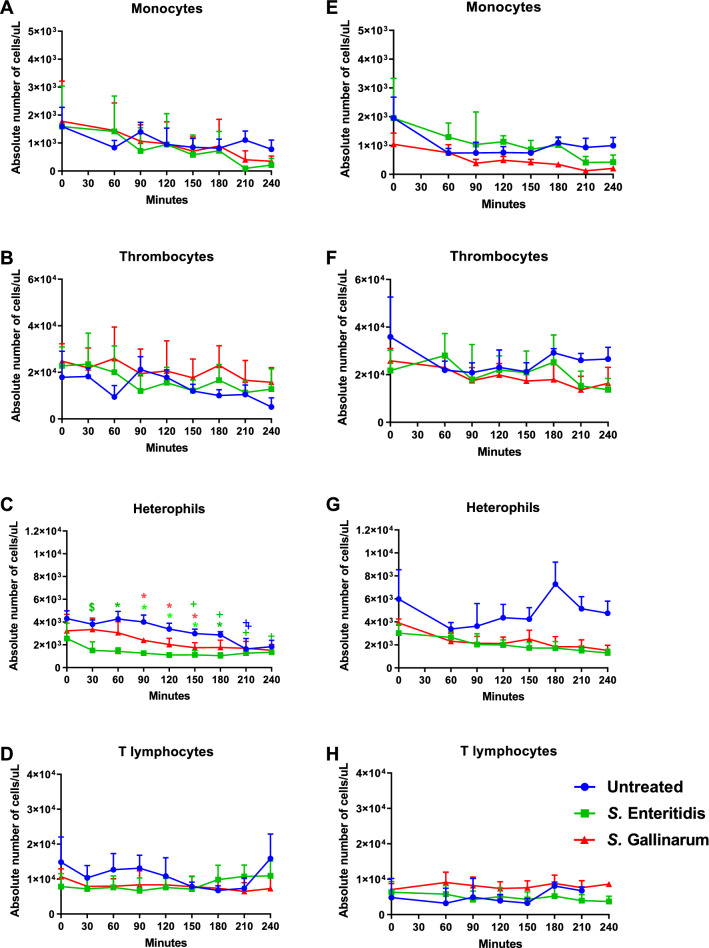
**Absolute numbers of immune cells in avian whole blood after *Salmonella* inoculation.** Avian whole blood from the chicken lines WLA (**A–D)**) and R11 (**E–H**) was incubated ex-vivo with *S.* Enteritidis and *S.* Gallinarum for 240 min. Numbers of viable monocytes, heterophils, thrombocytes and T lymphocytes were analysed every 30 min using flow cytometry. Data of six different donors per chicken line is presented as mean and SD. + indicates a significant difference compared to 0 min (*p* ≤ 0.05), * indicates a significant difference compared to the untreated blood sample (*p* ≤ 0.05), $ indicates a significant difference compared to *S.* Gallinarum (*p* ≤ 0.05). Colour represents respective infection status: Blue: non-infected control, Green: *S.* Enteritidis, Red: *S.* Gallinarum.

Flow cytometry was used to investigate whether SE and SG affected the absolute number of viable leucocytes in peripheral blood of the differently performing chicken lines WLA and R11 over time (Figure 2).

In non-treated samples, numbers of monocytes and thrombocytes slightly declined in both chicken lines over time of culture but with different magnitude. Lymphocyte numbers did not change. Numbers of heterophils were significantly reduced at 210 and 240 min in control samples of WLA.

After *Salmonella* inoculation, absolute numbers of heterophils decreased significantly in blood samples of WLA (Figure 2C). Compared to non-treated blood, significantly lower numbers (*p* ≤ 0.05) were seen between 60 and 180 min in SE- and between 90 and 150 min in SG-inoculated blood. Compared to 0 h, SE-inoculated blood samples showed a significant drop (*p* ≤ 0.05) in heterophil numbers between 150 and 240 min of incubation.

SE- and SG-treated blood of R11 showed lower numbers of heterophils than non-treated samples over time, but with no significant differences (Figure 2G).

Absolute numbers of monocytes, thrombocytes and T lymphocytes of *Salmonella*-treated samples were not significantly changed in comparison to non-treated blood of WLA and R11 over time.

### *Salmonella* survival in avian whole blood

**Figure 3:**
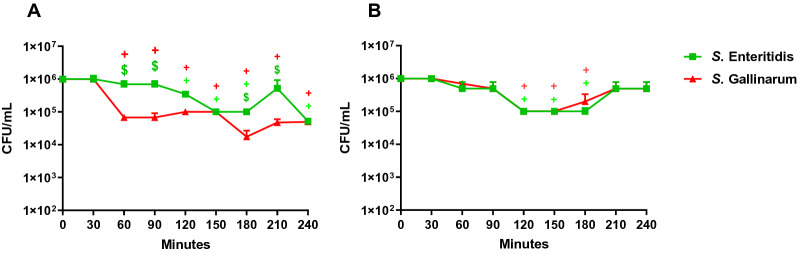
**Survival of *Salmonella* strains in avian whole blood.** Colony forming units of *S.* Enteritidis and *S.* Gallinarum were determined in the inoculum (0 min) and from samples taken every 30 min up to 240 min after inoculation with whole blood from WLA chickens (**A**) and R11 chickens (**B**). Data of six different donors per chicken line analysed in independent experiments is presented as mean and SD. + indicates a significant difference compared to 0 min (*p* ≤ 0.05), $ indicates a significant difference compared to *S.* Gallinarum (*p* ≤ 0.05). Colour represents the respective pathogen: Green: *S.* Enteritidis, Red: *S.* Gallinarum.

In order to verify the survival of SE and SG within peripheral blood of WLA and R11, the colony forming units (cfu) of the pathogens were calculated by microbiological plating every 30 min during the investigation period (Figure 3).

Chicken line-dependent differences in the survival rate of the *Salmonella* serovars were apparent. Both *Salmonella* serovars were better able to cope with the surrounding conditions in blood of R11 than of WLA. In blood of WLA, the number of SE and SG dropped (*p* ≤ 0.05) over time, with the lowest cfu count at 240 min (Figure 3A). At 60, 90, 180 and 210 min after *Salmonella* inoculation, significantly (*p* ≤ 0.05) lower amounts of SG than SE were found in blood of the high-performing chickens (WLA, Figure 3A). In blood of low-performing chickens (R11), the number of SE and SG did not change significantly from 0 to 240 min. However, there was a transient reduction of cfu of SE and SG between 120 and 180 min (Figure 3B).

### Association of host immune cells with *Salmonella* strains

**Figure 4:**
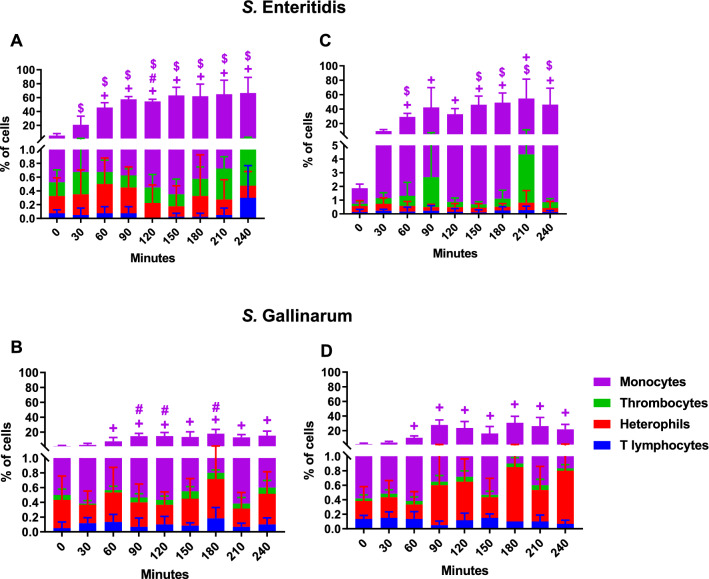
**Association of host cells with *Salmonella* strains in avian whole blood.** Association of different leucocyte subsets (monocytes, heterophils, thrombocytes and T lymphocytes) with GFP-transformed *S.* Enteritidis (**A**, **C**) and *S.* Gallinarum (**B**, **D**) in whole blood of WLA (**A**, **B**) and R11 chickens (**C**, **D**) was determined by flow cytometry and is presented as percentage of the leucocytes associated with *Salmonella* relative to the total host cell population in blood. Data of six different donors per chicken line analysed in independent experiments is presented as mean and SD. + indicates a significant difference compared to the 0 min time point (*p* ≤ 0.05), # indicates a significant difference compared to R11 chickens (*p* ≤ 0.05), $ indicates a significant difference compared to *S.* Gallinarum in the same chicken line (*p* ≤ 0.05).

To analyse the interaction of different immune cells with SE and SG, flow cytometry was used and the percentage of the leucocyte populations having direct contact with the GFP-expressing pathogens SE or SG examined (Figure 4).

Among the blood leucocytes of WLA and R11, only monocytes clearly associated with the *Salmonella* strains (Figures 4A–D). Irrespective of the chicken line used, significantly high numbers of monocytes interacted with *Salmonella* between 60 and 240 min of co-culture. Noticeably, in both chicken lines, significantly higher percentages of monocytes were in direct contact with SE than with SG (Figures 4A–D).

In blood of WLA and R11, less than 0.8% of heterophils and 4.3% of thrombocytes were associated with *Salmonella*. T lymphocytes showed hardly any interaction (lower than 0.3%) with *Salmonella*.

### Association of *Salmonella* strains with host immune cells

**Figure 5:**
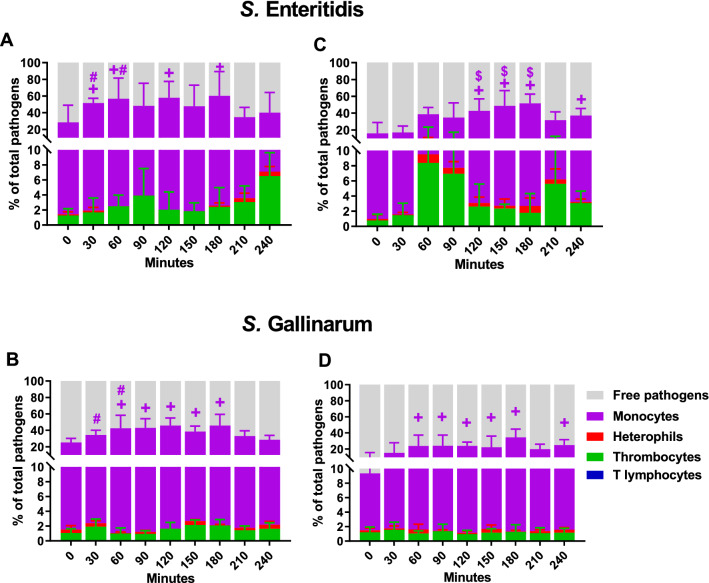
**Association of *Salmonella* strains with host cells in whole blood.** Association of GFP-transformed *S.* Enteritidis (**A**, **C**) and *S.* Gallinarum (**B**, **D**) with host cells (monocytes, heterophils, thrombocytes and T lymphocytes) in whole blood of WLA (**A**, **B**) and R11 chickens (**C**, **D**) was determined by flow cytometry and is presented as percentage of *Salmonella* associated with the host cell type relative to the total *Salmonella* population in the blood sample. Data of six different donors per chicken line analysed in independent experiments is presented as mean and SD. + indicates a significant difference compared to 0 min (*p* ≤ 0.05), # indicates a significant difference compared to R11 chickens (*p* ≤ 0.05), $ indicates a significant difference compared to *S.* Gallinarum in same chicken line (*p* ≤ 0.05).

To better understand the distribution of the pathogens in the blood compartments, such as serum and cells, we determined the percentages of SG and SE that were in direct contact with the different blood leucocytes and calculated the percentage of free pathogens in serum (Figure 5).

The results revealed high levels of free pathogens in blood serum, with the highest percentages occurring in SG-inoculated blood of the R11 line (Figure 5D).

The bacteria were mostly in contact with monocytes, followed by heterophils and thrombocytes. Whereas up to 80% of the *Salmonella* population were associated with monocytes at times, no more than 20% of the pathogens interacted with heterophils or thrombocytes. SE tended to associate in higher numbers with heterophils and thrombocytes than SG.

The percentages of *Salmonella* interaction with monocytes showed changes over time. The highest association was seen between 60- and 180-min cultivation time. At 30 and 60 min of blood culture, significantly more SE were associated with monocytes of WLA than with those of R11 (Figures 5A and C). In blood of R11, significantly more SE than SG had direct contact with monocytes between 120 and 180 min of incubation (Figures 5C and D).

### Transcription of immune-related avian proteins post infection

**Figure 6:**
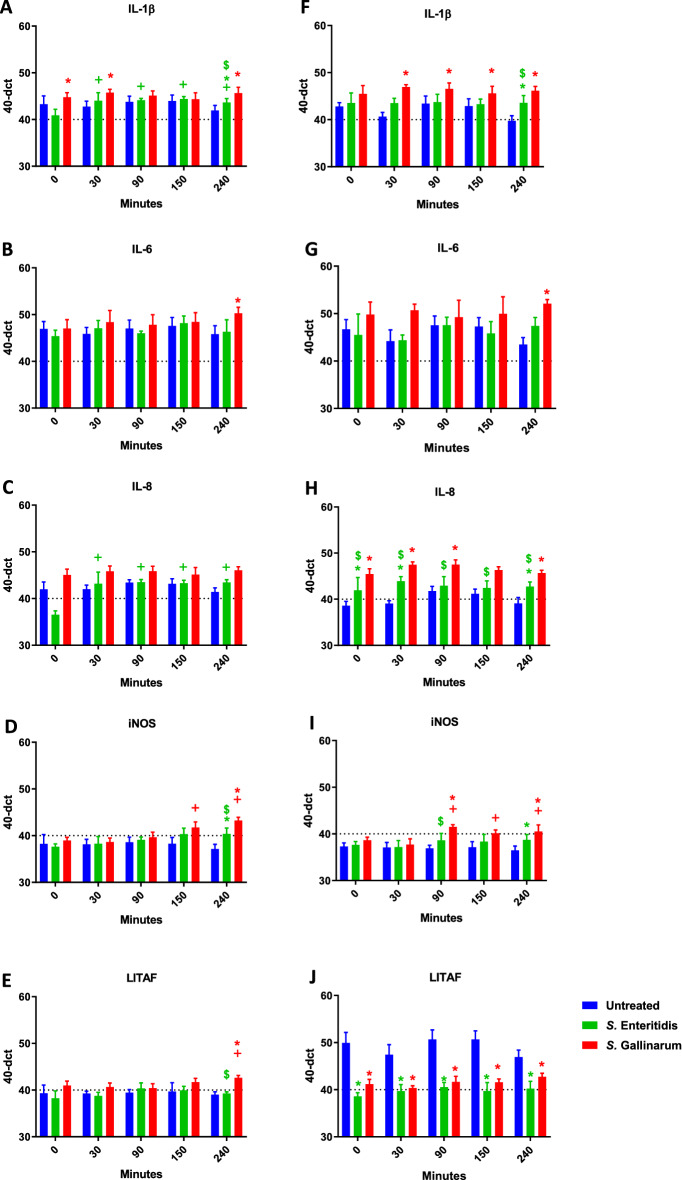
**Expression of immune related genes in whole blood after *Salmonella* inoculation.** The graphs represent the 40-ΔCt of gene expression in avian whole blood at the respective time points in blood of WLA (**A–E**) and R11 chickens (**F-J**). The data of six different donors per chicken line analysed in independent experiments is presented as mean and SD*.* + indicates a significant difference compared to 0 min (*p* ≤ 0.05), * indicates a significant difference compared to untreated blood (*p* ≤ 0.05), $ indicates a significant difference compared to *S.* Gallinarum (*p* ≤ 0.05). Colour represents the respective pathogen: Blue: control; Green: *S.* Enteritidis*;* Red: *S.* Gallinarum.

The *Salmonella* inoculation of avian peripheral blood of two differently performing laying hen lines led to a change of gene expression patterns of the immune mediators analysed in this study (Figure 6). With the exception of LITAF, blood samples of both chicken lines reacted to the pathogens with increased gene expression of all mediators investigated. The most pronounced up-regulation was seen for IL-8 expression in R11 hens (Figure 6H). Moreover, significantly higher IL-8 transcription levels were detected in SG- than in SE-inoculated samples of R11 at any time point examined in this study (Figure 6H). IL-6 mRNA and iNOS expression was up-regulated in SG-inoculated samples of R11 and WLA only at 240 min (Figures 6B, G, D, I). Compared to the non-treated control, iNOS transcription was increased in SE-stimulated samples of both chicken lines at 240 min and in SG-treated blood of R11 at 90 min (Figures 6D and I). Significant differences between SG and SE-treated samples were also present at these time points.

SG led to higher transcription levels of IL-1ß in blood immune cells from R11 and WLA with significant differences compared to SE at 240 min post infection.

There was a significant down-regulation of gene expression levels of LITAF in SG- and SE-stimulated blood of R11 at all time points examined in this study. In contrast, in WLA, no changes were seen, except for 240 min, where LITAF gene expression was significantly upregulated in SG-exposed samples when compared to both non-treated and SE-inoculated blood.

## Discussion

The aim of the study was to assess the interaction of the two differently host-adapted *Salmonella* serovars Enteritidis and Gallinarum with immune cells from peripheral blood of chicken lines showing different laying performance. Both *Salmonella* strains are virulent in chickens as shown in several in vivo assays [28].

The whole blood assay applied here was developed on the basis of assays already described [24, 29]. The method is most appropriate to detect and analyse host–pathogen interactions directly in avian whole blood and, thus, in a nearly natural environment.

In general, the nature of the host–pathogen interaction depends on the immune cells involved and is, at least for monocytes and heterophils, a dynamic process which can comprise different stages, as (i) attachment of *Salmonella* to the cell surface, (ii) phagocytosis by the immune cell, (iii) killing of *Salmonella* within or outside of immune cells with simultaneous loss of the green fluorescence signal or also (iv) escape of *Salmonella* from the immune cells. Similar to a recent study [24], we did not discriminate between attachment of pathogens to and phagocytosis by the immune cells in the present study. Others have described phagocytosis of *Salmonella* by avian blood cells, albeit a clear distinction between attachment of pathogens to and phagocytosis by immune cells was not proven by the authors [30]. Therefore, and because living *Salmonella* are able to enter immune cells actively, we refer to biological interaction or association between blood cells and *Salmonella* rather than to phagocytosis in the present study.

To assess the stability of the ex vivo avian whole blood model, we measured the absolute numbers of immune cells in blood with and without *Salmonella* exposure over 240 min of observation time. In our experiments, the changes in immune cell numbers were very moderate indicating a reasonably stable culture system. This finding is completely in line with a former study using blood samples of the same chickens lines together with human pathogens [24]. However, there was a significant reduction of heterophil numbers in *Salmonella*-inoculated blood of chickens with high egg production. The interaction of heterophils with pathogens is an important step in the response by the innate immune system. Chicken heterophils are able to phagocytose all sorts of pathogens followed by degranulation and production of an oxidative burst [31]. Whether there has been any uptake or killing of *Salmonella* by the peripheral heterophils in our study cannot be answered. Also, the question as to why heterophil numbers significantly dropped, although there was no direct association of *Salmonella* strains with the heterophils in our study, cannot be explained at the moment. Stress-induced mechanisms might be one possible explanation, but a very quick killing by the pathogens accompanied by a subsequent escape from FACS detection owing to the loss of membrane integrity and DAPI staining must also be considered. Intracellular bacteria and heterophil death after *Salmonella* stimulation has indeed been described by others [32].

While the heterophils in this study were barely associated with the bacteria, the monocytes showed pronounced interaction. Although we have not differentiated between *Salmonella* attachment to or phagocytosis by the monocytes, it can be expected that at least a certain proportion of the pathogens has been phagocytosed by monocytes. In a former study on human pathogens, the possibility and the assumption that the cell-pathogen association is indicative of or leads to phagocytosis was addressed by using a mathematical model [24]. In the case of *Salmonella* in the present study, their potential to actively enter host cells must be considered additionally when interpreting the results on the interaction with the monocytes.

Former studies demonstrated that an increased interaction of bacteria, such as *Escherichia coli* and *Staphylococcus aureus*, with avian blood monocytes coincided with a stronger decrease in monocyte numbers, suggesting killing by the pathogens [24]. For *Salmonella*, a potential to weaken avian monocytes through caspase activation and apoptosis has been suggested [17]. In the present study, monocyte numbers declined only slightly over time and significant differences between *Salmonella*-inoculated and -non-inoculated blood samples were not apparent. Thus, the slight reduction in monocytes numbers in our experiment might rather originate from stress factors during the culture than from direct killing by the bacteria.

Besides heterophils and monocytes/macrophages, thrombocytes have been identified as phagocytotic cell population in chickens [33]. It has already been accepted that thrombocytes are immunologically active cells and able to respond to bacteria or their products. Using the K1 antibody, we found an association of thrombocytes and *Salmonella*, which, with up to 4.3% of thrombocytes at 210 min of co-cultivation, was especially pronounced for SE in the blood of R11 hens. In an older study, percentages of more than 14% of the thrombocyte population were shown to have direct contact to *Salmonella* after a 60-min incubation at 37 °C. However, the authors used isolated thrombin-stimulated thrombocytes for their investigations [34]. Even though a phagocytotic capacity has been postulated for thrombocytes of non-mammalian vertebrates [35], the question whether the avian thrombocytes of our study were able to engulf the *Salmonella* serovars must remain open. However, thrombocytes express Toll-like receptors (TLR) [36–38], which can trigger the release of proinflammatory cytokines, such as IL-6, IL-1β and IL-8 [36–39] or the enzyme iNOS [36]. Since thrombocytes constitutively express TLR4 [37], activation of these cells by the *Salmonella* strains of our study is conceivable. Indeed, the treatment of thrombocytes with LPS results in an increase of IL-1β and IL-6 transcription [36, 37]. Therefore, a contribution of thrombocytes to the IL-1β-gene expression found in our study cannot be excluded.

In the present study, direct interaction of *Salmonella* with T lymphocytes was nearly absent. For CD4^+^ and CD8^+^ T cells, direct blocking of their proliferation due to down regulation of the T-cell-receptor-β-chain expression by *Salmonella* Typhimurium has been described [40, 41]. The enzyme L-asparaginase II has been shown to be responsible for this inhibitory effect as well as for the blockade of cytokine production by these cells after exposure to *Salmonella* [40, 41]. In addition, *Salmonella* can also indirectly stimulate T cells via TLRs or other pattern recognition receptors (PRRs) on mononuclear phagocytes, and the resulting cytokines produced can prime T cells [42, 43]. The extent to which such mechanisms may, however, have played a role in our study must be revealed by more detailed investigations in the future.

Bacteria can be cleared from blood circulation, but the mechanisms have not been clarified yet [44]. In the present study, a decrease in *Salmonella* numbers was detectable in blood of both chicken lines, though with different progressions. After 4 h of co-culture, we found a slightly better *Salmonella* survival in blood cultures of the low-performing R11 line than of the high-performing WLA line. The fluctuating numbers of *Salmonella* in the cultures additionally found during the incubation time speaks well for a process by which the bacteria try to adapt to the new, unknown environment. In blood, the *Salmonella* are exposed to a variety of immunologic defence mechanisms in form of serum constituents and immune cells. Chicken serum alone, however, seems not to kill or inhibit *Salmonella* [30]. In our samples, antimicrobial peptides released from heterophils following degranulation might have killed *Salmonella*. Formation of extracellular traps by *Salmonella*-stimulated heterophils has already been demonstrated [32] and could have been responsible for the reduction in the number of pathogens in our experiment. The clearance of the bloodstream from bacteria may also have been performed by oxycytosis: erythrocytes catch bacteria by electric charge attraction and kill them by the oxygen released from oxyhaemoglobin [44].

The outcome of an infection is particularly dependent on the interactions between the pathogens with their differences in virulence and the assorted immune cells with their distinct functions. On the one hand, monocytes are specialized to recognize, engulf and kill all kinds of pathogens. On the other hand, *Salmonella* are able to actively invade host cells and multiply intracellularly. Our results demonstrated a higher interaction of SE than SG with avian monocytes. Therefore, we suggest that the non-host-adapted SE possesses a higher activity and virulence in blood compared to the host-restricted SG. This assumption is supported by other studies, which have shown a weaker adhesion/invasion ability of SG than SE by using both chicken and human epithelial cell lines [18, 45]. A hypothesis is that the lower adhesion capacity of SG compared to SE hampers the intimate contact between SG and the host cell and thus impairs the injection of type three secretion system one (T3SS-1) effectors, which is an essential step for entering the host cell. It has also been postulated that the weak invasiveness of SG is related to mutations in *Salmonella* pathogenicity island one (SPI-1) genes [45].

The data presented here show that cells of avian peripheral blood are able to respond to the differently host-adapted *Salmonella* serovars and to produce a serovar-specific pattern of immune-related mediators. The transcriptional upregulation of pro-inflammatory cytokines and chemokines shown in this study indicates a fast defence reaction in blood. It may be concluded that the exclusive interaction of *Salmonella* with phagocytes and not with T lymphocytes found here could have triggered gene expression of the immune regulators in peripheral blood. How far soluble bacterial factors, such as LPS, might have been responsible for triggering the transcription response cannot be answered. Our results on increased transcription of immune-related mediators in blood are consistent with previous studies showing an induction of pro-inflammatory cytokines after bacterial stimulation of human or avian blood [24, 46]. The more pronounced upregulation of IL-8 in SG- than in SE-infected R11 chickens underlines the lower cytotoxicity of SG compared to SE [45, 47] and suggests a stronger immune response of blood cells from R11 compared to WLA chickens. However, the number of immune-related genes analysed in this study is rather small and included only a selected choice of effectors mainly produced by monocytes. In future studies, a broader range of cytokines or a more global transcriptome or proteome analysis could give much more information on the specific response of the immune cells involved.

The extent to which stress, such as high laying performance of hens, influences the effectiveness of the immune response against *Salmonella* is still largely unclear. For this reason, we used two White Leghorn chicken lines differing in their egg laying performance [48]. The high performing genotype shows approximately a 30% increased laying intensity compared to the low performing chickens along with some functional differences [19, 48]. It has already been shown that high egg-laying chickens have reduced capacity to compensate to unexpected climate changes and a high reduction in their body weight due to a limited diet as compared to the low performing chickens [19, 48]. In the present study, we found a lower *Salmonella* survival rate in blood cultures of the high-performing WLA line than of the low-performing R11 line. Against viral infections, high performing chickens exhibit higher stress than low performers, thereby also affecting the immune response [49]. A lot of work has been done to find genetic reasons for the different susceptibility of specific chicken lines against different *Salmonella* serovars [50]. Genetic resistance of chickens to systemic salmonellosis seems to be associated with genomic regions carrying the candidate genes NRAMP1 (natural resistance-associated macrophage protein, now SLC11A1), MHC, TLR4 and the quantitative trait locus SAL1 [50].

In summary, following bacterial inoculation of the whole blood of two chicken lines with different laying performance, we detected an interaction of the bacteria with monocytes, whereby SE showed a more pronounced interaction compared to SG. Besides the drop in heterophil numbers in *Salmonella*-inoculated blood of WLA chickens, a decrease of bacterial numbers was found over the time of incubation. In response to the bacterial load, immune cells of blood were also able to react by transcription of immune mediators such as IL-8. It should be noted that our study focused only on the cellular association of SE and SG. The real nature of the host–pathogen interaction, which would include cellular attachment and phagocytosis of SE and SG, was not addressed in the present work and needs to be investigated in future studies.

In general, our results provide early insights into the interaction of *Salmonella* strains with different immune cell populations in avian whole blood, demonstrating *Salmonella* strain-specific and chicken line-dependent differences. Further studies will be necessary to elucidate the functional importance of the associations observed as well as the underlying molecular mechanisms.

## Data Availability

The datasets generated and analysed during the current study are available from the corresponding author on reasonable request.

## Abbreviations

BD: Becton Dickinson
cfu: colony forming units
DAPI: 4’,6-Diamidin-2-phenylindol
FLI: Friedrich-Loeffler-Institut
GFP: green fluorescent protein
LPS: lipopolysaccharide
MHC: major histocompatibility complex
Mɸ: monocyte
OD: optical density
PBS: phosphate-buffered saline
rpm: revolutions per minute
*S*.: *Salmonella*
SE: *Salmonella* Enteritidis 147
SG: *Salmonella* Gallinarum SG9
SSC: sideward-scatter
TLR: Toll-like receptor
